# Involvement of JNK and Caspase Activation in Hoiamide A-Induced Neurotoxicity in Neocortical Neurons

**DOI:** 10.3390/md13020903

**Published:** 2015-02-10

**Authors:** Zhengyu Cao, Xichun Li, Xiaohan Zou, Michael Greenwood, William H. Gerwick, Thomas F. Murray

**Affiliations:** 1 State Key Laboratory of Natural Medicines and Jiangsu Provincial Key Laboratory for TCM Evaluation and Translational Development, School of TCM, China Pharmaceutical University, Nanjing 211198, China; E-Mails: Xichun_li@163.com (X.L.); imzouxiaohan@163.com (X.Z.); 2 Department of Pharmacology, School of Medicine, Creighton University, Omaha, NE 68178, USA; E-Mail: michael.greenwood@unmc.edu; 3 Center for Marine Biotechnology and Biomedicine, Scripps Institution of Oceanography and Skaggs School of Pharmacy and Pharmaceutical Sciences, University of California, San Diego, La Jolla, CA 92093, USA; E-Mail: wgerwick@ucsd.edu

**Keywords:** apoptosis, caspase, c-Jun *N*-terminal kinase, hoiamide A, necrosis

## Abstract

The frequent occurrence of *Moorea producens* (formerly *Lyngbya majuscula*) blooms has been associated with adverse effects on human health. Hoiamide A is a structurally unique cyclic depsipeptide isolated from an assemblage of the marine cyanobacteria *M. producens* and *Phormidium* *gracile*. We examined the influence of hoiamide A on neurite outgrowth in neocortical neurons and found that it suppressed neurite outgrowth with an IC_50_ value of 4.89 nM. Further study demonstrated that hoiamide A stimulated lactic acid dehydrogenase (LDH) efflux, nuclear condensation and caspase-3 activity with EC_50_ values of 3.66, 2.55 and 4.33 nM, respectively. These data indicated that hoiamide A triggered a unique neuronal death profile that involves both necrotic and apoptotic mechanisms. The similar potencies and similar time-response relationships between LDH efflux and caspase-3 activation/nuclear condensation suggested that both necrosis and apoptosis may derive from interaction with a common molecular target. The broad-spectrum caspase inhibitor, Z-VAD-FMK completely inhibited hoiamide A-induced neurotoxicity. Additionally, hoiamide A stimulated JNK phosphorylation, and a JNK inhibitor attenuated hoiamide A-induced neurotoxicity. Collectively, these data demonstrate that hoiamide A-induced neuronal death requires both JNK and caspase signaling pathways. The potent neurotoxicity and unique neuronal cell death profile of hoiamide A represents a novel neurotoxic chemotype from marine cyanobacteria.

## 1. Introduction

Marine cyanobacteria are prolific producers of structurally novel and biologically active natural products and are especially rich in metabolites with toxic properties [1,2,3,4]. *Moorea producens* (formerly *Lyngbya majuscula*) is a pantropical marine cyanobacterium, blooms of which have been occurring for decades around the world, particularly in Florida’s Gulf Coastal region of Sanibel Island and the east coast of Queensland, Australia. These blooms of *M. producens* are reported to have adverse effects on both human populations and domestic animals [5,6], including respiratory irritation, eye inflammation, severe contact dermatitis, gastrointestinal distress as well as fever and headache symptoms [5,7,8,9]. Chemical investigations of *M. producens* have revealed several classes of structurally unique secondary metabolites that are toxic to mammalian cells [7,10,11,12,13,14,15,16,17,18,19,20,21]. However, the modes of action of these natural toxins are less studied.

Hoiamide A is a novel bioactive cyclic depsipeptide isolated from an environmental assemblage of the marine cyanobacteria *Moorea producens* and *Phormidium gracile* collected in Papua New Guinea [22]. This stereochemically complex metabolite possesses a highly unusual structure that likely derives from a mixed peptide-polyketide biogenetic origin, and includes a peptidic section featuring a ketide-extended and *S*-adenosyl methionine modified isoleucine moiety, a triheterocyclic fragment bearing two-methylated thiazolines and one thiazole, and a highly oxygenated and methylated C15-polyketide substructure [22]. After discovering hoiamide A, the structurally related analogs, hoiamides B–D, were purified from either *Symploca* sp. or an assemblage of *Symploca* sp. and *Oscillatoria cf.* sp. [23,24]. Due to its unique and intriguing structure, hoiamide C became the target of total organic synthesis; this was successfully accomplished in 2011 [25].

In primary cultures of neocortical neurons, we have shown that pure hoiamide A stimulated sodium influx with a low micromolar EC_50_ value. The triggered sodium influx was abrogated by co-application of the sodium channel blocker tetrodotoxin (TTX), suggesting that hoiamide A may act as a voltage gated sodium channel (VGSC) activator [22]. Direct evidence of hoiamide A interaction with VGSCs was derived from its ability to inhibit [^3^H]batrachotoxin binding to VGSCs [22]. Further examination of hoiamide A’s effects on sodium influx demonstrated that hoiamide A is a partial agonist of neurotoxin site 2 on the voltage-gated sodium channel [22]. In addition to their action on the VGSCs, hoiamide A and hoiamide B suppressed spontaneous Ca^2+^ oscillations in primarily cultures of cortical neurons at sub-micromolar concentrations. This latter effect was independent of modification of VGSC activity [23]. In contrast, the linear analog, hoiamide C, was inactive in disrupting spontaneous Ca^2+^ oscillations [23]. Another linear analog, hoiamide D, was found to be an inhibitor of p53/MDM2 interaction at micromolar concentrations, an attractive target for anti-cancer drug development [24]. The hoiamides therefore appear to interact with several biologically significant molecular targets with distinct affinities.

Sodium channel activators have been shown to stimulate neurite outgrowth through enhancement of NMDA receptor function in neocortical neurons [26,27]. In the present study we explored the influence of hoiamide A on neurite outgrowth in neocortical neurons. In contrast to the neurite outgrowth stimulated by sodium channel activators, hoiamide A produced a concentration-dependent neurite retraction in neocortical neurons having an IC_50_ value of 4.89 nM with a 95% Confidence Interval (95% CI) of 1.14–20.9 nM. Additional studies demonstrated that hoiamide A increased LDH efflux, produced nuclear condensation and stimulated caspase-3 activity all with low nanomolar potency. These data indicate that hoiamide A triggers a unique profile of neuronal death in neocortical neurons that involves both necrotic and apoptotic mechanisms. The actions of hoiamide A on neurite retraction and neurotoxicity were three orders of magnitude more potent than its action on sodium channels, thus excluding VGSCs as the molecular target responsible for neurotoxicity. Further pharmacological evaluation demonstrated that hoiamide A-induced neurotoxicity was dependent on both caspase and JNK activation. 

## 2. Results 

### 2.1. Hoiamide A Produces Neurite Retraction in Neocortical Neurons

**Figure 1:**
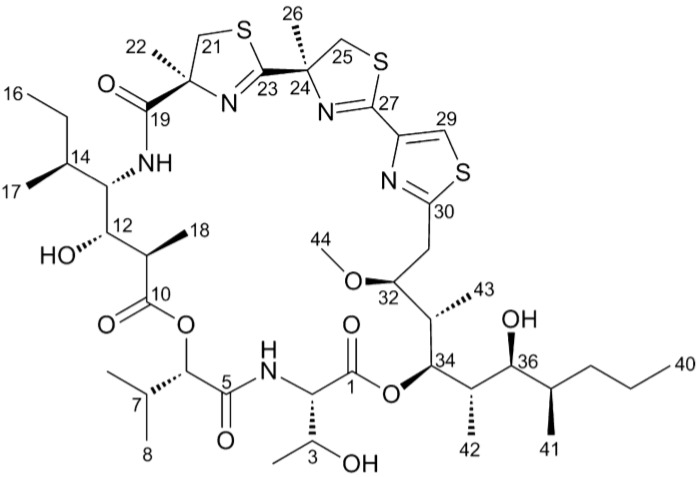
Chemical structure of Hoiamide A.

**Figure 2:**
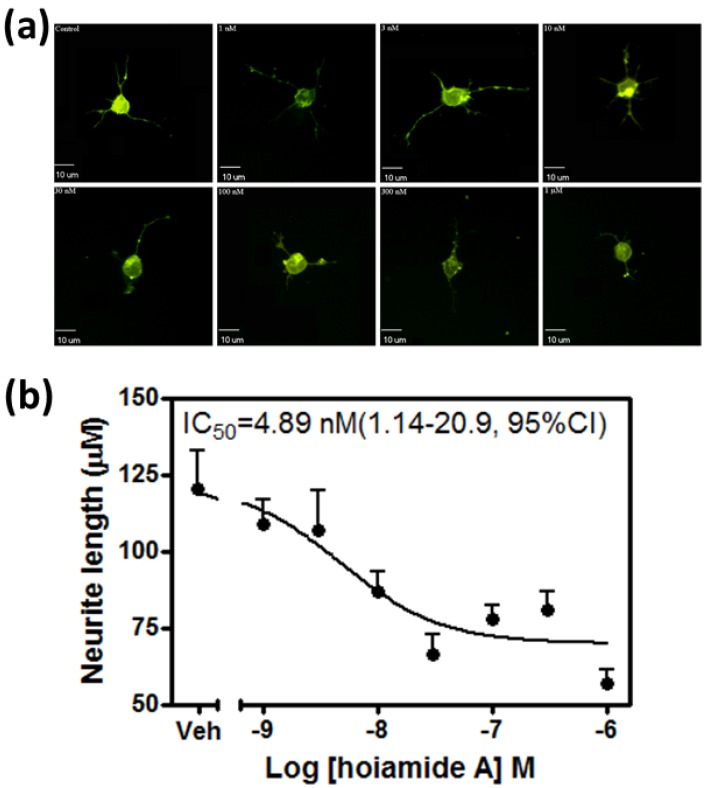
Hoiamide A induced retraction of neurites in neocortical neurons. (**a**) Representative images of DiI-loaded immature neocortical neurons treated with various concentrations of hoiamide A for 24 h; (**b**) Concentration-response relationship for hoiamide A on neurite retraction at 24 h post plating. Experiments were performed three times and each point represents the mean value derived from analysis of 25–30 neurons.

The structure of hoiamide A was shown in Figure 1. Given the partial agonist activity of hoiamide A at neurotoxin site 2 on VGSCs [22] and the previously demonstrated stimulation of neurite outgrowth by VGSC activators such as PbTx-2 and antillatoxin [26,27], we evaluated the influence of hoiamide A on neurite outgrowth in neocortical neurons. Three hours post plating the cells were treated with vehicle (0.1% DMSO) and different concentrations of hoiamide A for 24 h. The cells were then labelled with DiI dye using the Helios Gene Gun System and the images were taken on an Olympus IX71 fluorescent microscope. Rather than an increase in neurite outgrowth, hoiamide A produced a concentration-dependent neurite retraction in immature neocortical neurons (Figure 2). The IC_50_ value revealed by non-linear regression analysis was 4.89 nM with a 95% Confidence Interval (95% CI) of 1.14–20.9 nM. 

### 2.2. Hoiamide A Produces LDH Efflux in Neocortical Neurons

**Figure 3:**
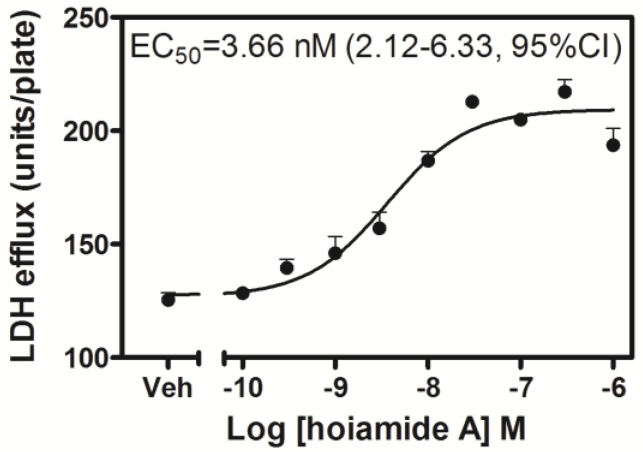
Neurotoxic concentration-response relationship for hoiamide A induced LDH efflux in neocortical neurons exposed for 24 h. Individual points represent the mean ± S.E.M. from three separate experiments performed in triplicate.

**Figure 4:**
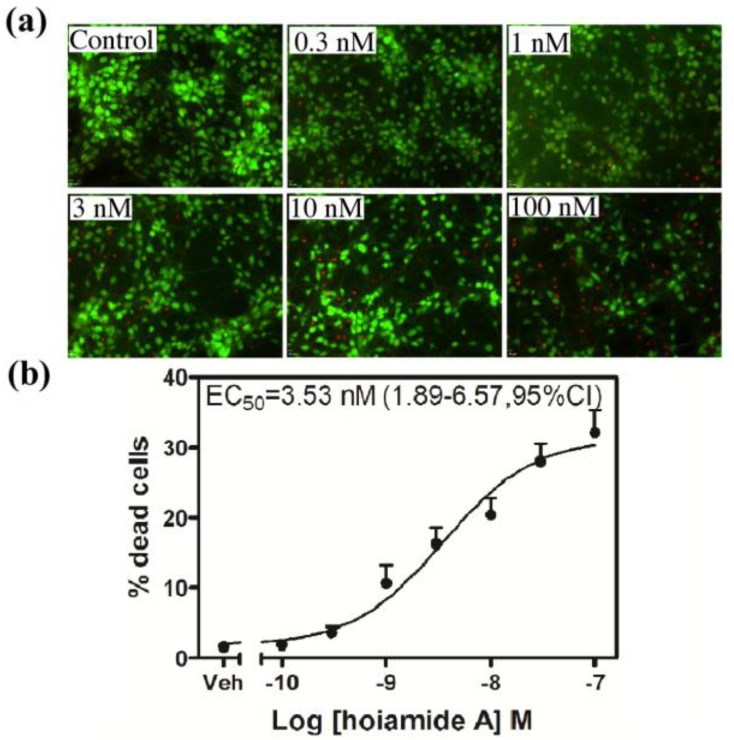
Hoiamide A-induced neurotoxicity. (**a**) Representative images for FDA and PI double-stained neocortical neurons treated with various concentrations of hoiamide A for 24 h; (**b**) Concentration-response relationship for hoaimide A-induced neuronal death visualized by PI staining. Experiments were performed three times each in triplicate.

Neocortical neurons exposed to hoiamide A (30 nM) for 8, but not 2 h displayed significant LDH efflux (data not shown). After a 24 h duration of exposure, hoiamide A produced robust neuronal death as reflected in significantly elevated levels of LDH efflux. This hoiamide A neurotoxicity was concentration-dependent with an EC_50_ value of 3.66 nM (1.12–6.33, 95% CI) (Figure 3). To further confirm hoiamide A-induced neurotoxicity, neocortical neurons were treated with various concentrations of hoiamide A for 24 h and then stained with fluorescein diacetate (FDA) and propidium iodide (PI). As shown in Figure 4a, the percentage of live cells (green, FDA fluorescence) gradually decreased whereas the percentage of dead cells (red, PI fluorescence) gradually increased with increased concentrations of hoiamide A. Figure 4b depicts the concentration-response curve for hoiamide A-induced neurotoxicity as determined by PI measurements, and was consistent with the results of the LDH assay [EC_50_ = 3.53 nM (1.89–6.57, 95% CI)]. 

### 2.3. Hoiamide A Stimulates Caspase-3 Activation in Neocortical Neurons

**Figure 5:**
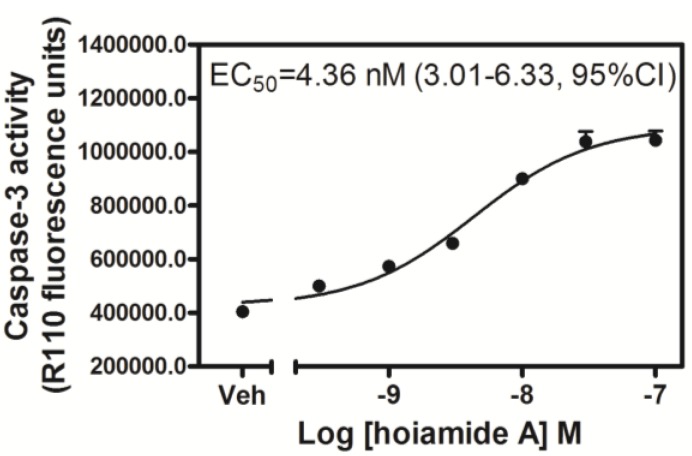
Concentration-response relationship for hoiamide A-induced caspase-3 activation. Experiments were repeated in three independent cultures, each in triplicate.

Apoptosis and necrosis are generally recognized as two distinct pathways of cell death. Given that LDH efflux represents a useful assay for necrotic cell death, we next investigated whether hoiamide A was capable of producing apoptotic cell death in neocortical neurons. Caspase proteases play a critical role in apoptotic neuronal death and the activation of caspases has been considered to be an early marker of apoptotic cell death. We accordingly tested the influence of hoiamide A on caspase-3 activity in neocortical neurons. Caspase-3 activity was significantly enhanced after 8, but not 2 h of treatment with hoiamide A (data not shown). As depicted in Figure 5, treatment with hoiamide A for 24 h produced a robust and concentration-dependent stimulation of caspase-3 activity with an EC_50_ value of 4.36 nM (3.01–6.33, 95% CI). 

### 2.4. Hoiamide A Produces Nuclear Condensation in Neocortical Neurons 

**Figure 6:**
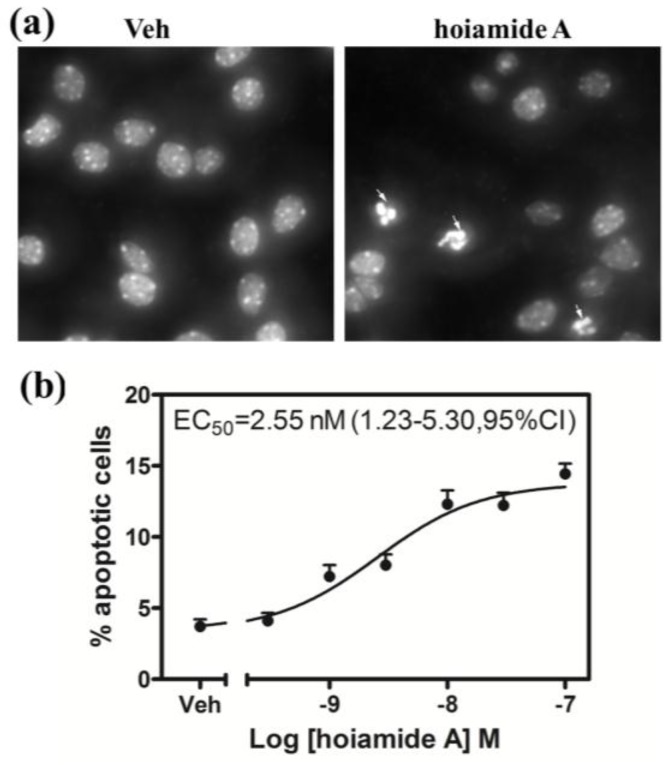
Hoiamide A stimulation of nuclear condensation in neocortical neurons. (**a**) Representative neocortical neurons after exposure to 0.1% DMSO for 24 h; (**b**) Representative neocortical neurons exposed to 30 nM hoiamide A for 24 h. Neocortical neuron condensed nuclei were revealed with Hoechst 33342 dye staining (indicated with arrows); (**c**) Quantification of concentration-response relationship for hoiamide A-induced nuclear condensation. Experiments were repeated in three independent cultures, each in triplicate.

An additional characteristic of apoptotic cell death is the morphological change that occurs to nuclear chromatin that manifests as nuclear condensation. We therefore evaluated the effects of hoiamide A on nuclear morphology in neocortical neurons. Neocortical neurons displayed significant nuclear morphological changes after 24 h exposure to hoiamide A (Figure 6a). Hoiamide A produced a robust and concentration-dependent stimulation of nuclear condensation and fragmentation in neocortical neurons with an EC_50_ value of 2.55 nM (1.23–5.30, 95% CI) (Figure 6b). 

### 2.5. Tetrodotoxin (TTX) is without Effect on Hoiamide A-Induced Neurotoxicity 

**Figure 7:**
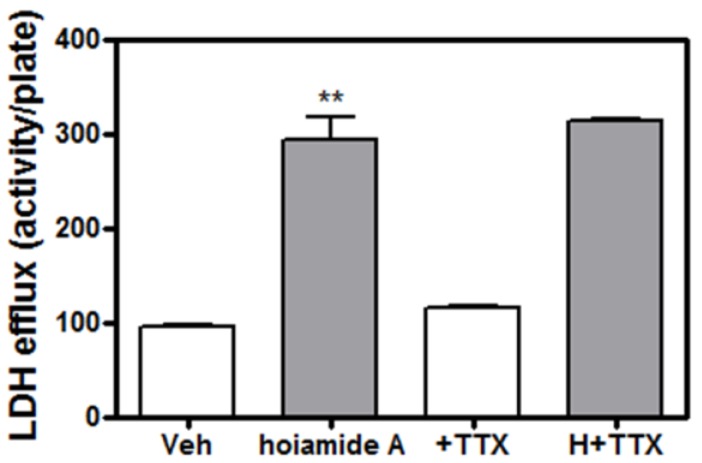
Lack of influence of TTX on hoiamide A-induced LDH efflux. TTX was without protective effect on hoiamide A-induced LDH efflux. These data were obtained from two independent cell cultures each performed in triplicate (** *p* < 0.01, hoiamide A *vs.* vehicle).

Given the previous demonstration that hoiamide A is a low affinity VGSC partial agonist at neurontoxin site 2 [22], we next determined whether hoiamide A-induced neurotoxicity in neocortical neurons involved activation of VGSCs. Neocortical neurons were exposed to TTX, an inhibitor of VGSCs, for 1h prior to the addition of 30 nM hoiamide A. This pretreatment with TTX did not afford a protective effect against hoiamide A-induced LDH efflux (Figure 7).

### 2.6. Caspase Inhibitor, Z-VAD-FMK, Antagonizes Hoiamide A-Induced Neurotoxicity

**Figure 8:**
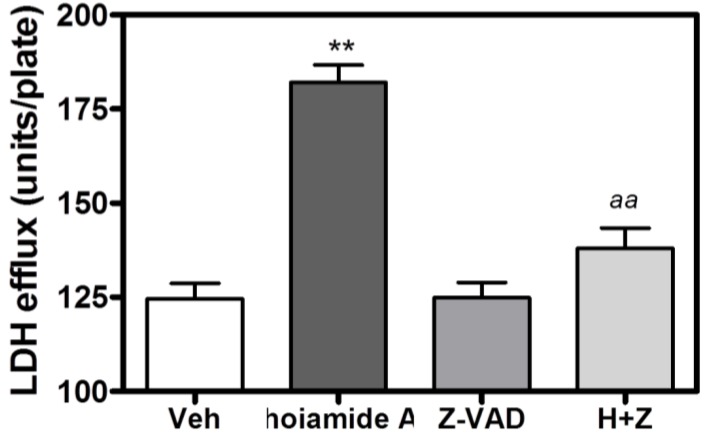
Effect of Z-VAD-FMK (100 μM) on hoiamide A-induced LDH efflux. These data were obtained from two independent cell cultures performed in triplicate (** *p* < 0.01, hoiamide A *vs.* vehicle; *aa*, *p* < 0.01, hoiamide A + Z-VAD-FMK *vs.* hoiamide A).

**Figure 9:**
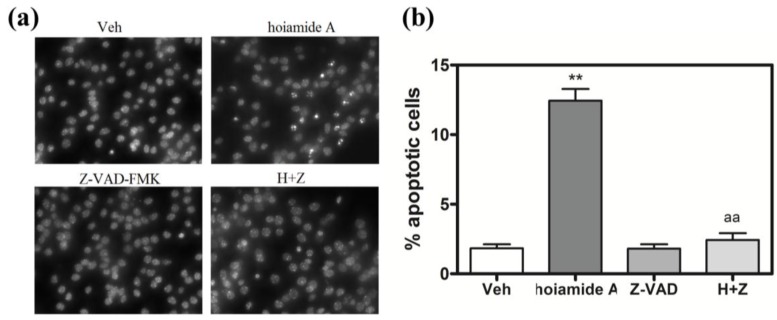
Effect of Z-VAD-FMK (100 μM) on hoiamide A-induced nuclear condensation. (**a**) Representative Hoechst 33342 stained images of neocortical neurons exposed to 0.01% DMSO or hoiamide A (30 nM) in the absence or presence of the caspase inhibitor Z-VAD-FMK (100μM); (**b**) Z-VAD-FMK (100 μM) suppressed hoiamide A-induced nuclear condensation. These data were obtained from two independent cell cultures each performed in quadruplicate (** *p* < 0.01, hoiamide A *vs.* vehicle; *aa*, *p* < 0.01, hoiamide A + Z-VAD-FMK *vs.* hoiamide A).

Given that hoiamide A stimulated caspase-3 activity, we evaluated the role of a broad-spectrum caspase inhibitor, Z-VAD-FMK, on hoiamide A-induced neurotoxicity in neocortical neurons. Neocortical neurons were pretreated with Z-VAD-FMK (100 μM) for 1 h prior to addition of 30 nM hoiamide A. Pre-treatment with Z-VAD-FMK produced a nearly complete inhibition of both hoiamide A-induced LDH efflux (Figure 8) and nuclear condensation (Figure 9).

### 2.7. Lack of Effect of MK-8*0*1, Nifedipine, NBQX and KBR-7943 on Hoiamide A-Induced Neurotoxicity in Neocortical Neurons

To further explore the molecular mechanisms responsible for hoiamide A-induced neurotoxicity, we next evaluated the roles of NMDA receptors, L-type Ca^2+^ channels, α-amino-3-hydroxy-5-methyl-4-isoxazolepropionic acid (AMPA) receptors and the Na^+^-Ca^2+^ exchanger as targets for hoiamide A-induced neurotoxicity. Application of an NMDA receptor antagonist (MK-801, 1 μM), L-type Ca^2+^ channel antagonist (nifedipine, 1 μM), AMPA receptor antagonist (NBQX, 1 μM) or the Na^+^-Ca^2+^ exchanger reverse mode inhibitor (KBR-7943, 3 μM) for 1 h prior to the addition of hoiamide A (30 nM) had no effect on hoiamide A-induced LDH efflux (Supplementary Figure S1). 

### 2.8. The JNK Inhibitor, SP 6*00*125, Protects Neocortical Neurons from Hoiamide A-Induced Neurotoxicity

**Figure 10:**
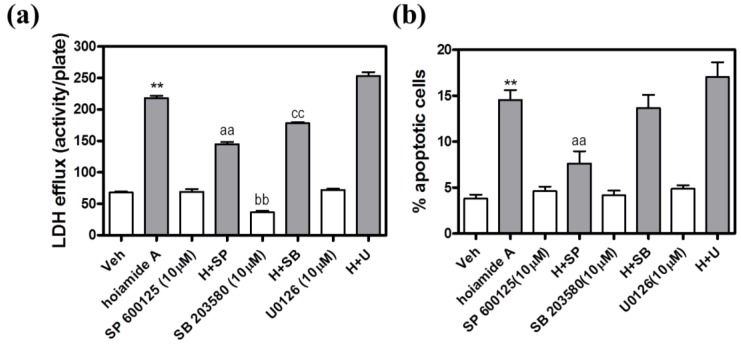
(**a**) Effect of MAP kinase inhibitors on hoiamide A-induced LDH efflux. These data were obtained from two independent cell cultures each performed in triplicate (** *p <* 0.01, hoiamide A *vs.* vehicle; aa, *p <* 0.01, hoiamide A (H)+SP *vs.* hoiamide A; bb, *p <* 0.01, SB 203580 *vs.* vehicle; cc, *p <* 0.01, H+SB *vs.* hoiamide A); (**b**) Effect of MAP kinase inhibitors on hoiamide A (30 nM)-induced nuclear condensation. These data were obtained from two independent cell cultures each performed in triplicate. (** *p <* 0.01, hoiamide A *vs.* vehicle; aa, *p <* 0.01, H + SP *vs.* hoiamide A).

Mitogen activated protein (MAP) kinases are involved in neuronal cell death induced by multiple mechanisms. We therefore evaluated the effect of MAP kinase inhibitors on hoiamide A-induced neurotoxicity. Inhibition of the extracellularly regulated kinase (ERK) activity by U0126 (10 μM) had no protective effects against either hoiamide A-induced LDH efflux or nuclear condensation (Figure 10). The p38 kinase inhibitor, SB 203580 (10 μM), produced a modest inhibition of hoiamide A-induced LDH efflux; however, this effect was apparently due to inhibition of basal LDH efflux. Pretreatment with SB 203580 (10 μM) was without effect on either basal or hoiamide A-induced nuclear condensation. To evaluate the role of the JNK cascade in the neurotoxic effect of hoiamide A (30 nM), we treated neocortical neurons with the JNK inhibitor, SP 600125 (10 µM). Pretreatment with SP 600125 produced a statistically significant protective effect against hoiamide A-induced LDH efflux (49% decrease) as well as chromatin condensation (65% decrease) (Figure 10). 

### 2.9. Hoiamide A Stimulates JNK Phosphorylation on Neocortical Neurons

**Figure 11:**
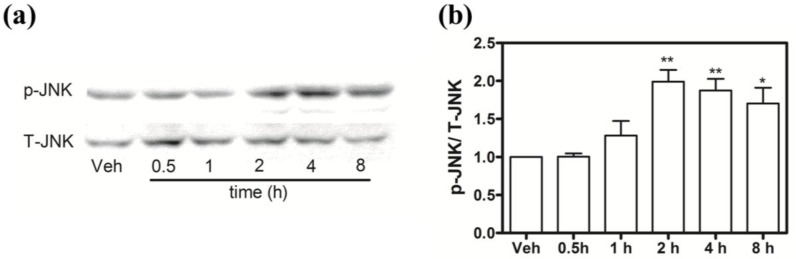
Influence of hoiamide A on JNK. (**a**) Representative western blot for the influence of hoiamide A (30 nM) on JNK phosphorylation; (**b**) Quantification of JNK phosphorylation after exposure of neocortical cells to hoiamide A (30 nM) as a function of time. These data were pooled from four independent experiments (** *p <* 0.01; * *p <* 0.05, hoiamide A *vs.* vehicle).

Given the protective effects of the JNK inhibitor SP 600125 against hoiamide A-induced neurotoxicity, we evaluated the effect of hoiamide A on JNK phosphorylation. Exposure to hoiamide A (30 nM) produced a significant increase in JNK phosphorylation that peaked at 2 h (Figure 11). The anti-JNK antibody usually recognizes a doublet in many cell lines. However, in our neocortical cultures it appears that only one isoform is preferentially expressed and activated.

## 3. Discussion

Hoiamide A is a novel bioactive cyclic depsipeptide isolated from an environmental assemblage of the marine cyanobacteria *M. producens* and *P. gracile* collected in Papua New Guinea [22]. Hoiamide A was isolated in substantial quantities from this assemblage (0.1% of the total dry weight, 150 mg from 150 g dry weight cyanobacteria) [22]. In the present study we have demonstrated that at low nanomolar concentrations hoiamide A significantly reduced neurite outgrowth, increased LDH efflux and enhanced both caspase activation and nuclear condensation. These data demonstrate that hoiamide A is a potent neurotoxin. Blooms of *M. producens* (formerly, *Lyngbya majuscula*) have been reported to be associated with adverse effects on both human populations and domestic animals [5,6] including fever and headache consistent with a neurologic effect [5,7,8,9]. Whether hoiamide A is also responsible for adverse health effects observed during blooms of *M. producens* will require further examination.

During the process of apoptosis, cells undergo several characteristic morphological changes such as cell shrinkage, nuclear/chromatin condensation, membrane blebbing and formation of apoptotic bodies [28,29,30]. One of the mechanisms responsible for apoptotic morphological changes is the activation of proteases, such as those of the caspase family, whose activation is considered to be an early marker of apoptosis [31,32]. We demonstrate here that hoiamide A treatment produced neuronal apoptosis as evidenced by both nuclear condensation and stimulation of caspase-3 activity in neocortical neurons; this occurred with similar potency to the observed hoiamide A-induced LDH efflux induced. These similar nanomolar potencies and time-response relationships between LDH efflux and caspase activation/nuclear condensation are consistent with a common molecular target for both pharmacological actions. 

An interesting feature of hoiamide A-induced neurotoxicity is that it includes both necrotic and apoptotic processes in neocortical neurons, and that these distinct types of cell death appear to occur simultaneously. The hybrid cell death is similar to that caused by another type of neurotoxin, the azaspiracids [33]. Azaspiracid-1 (AZA-1) produced neurotoxicity through both necrosis and apoptosis [33]. Whether the targets of hoiamide A and AZAs are similar is presently unknown; however, a critical difference between hoiamide A and AZA-1 induced neurotoxicity is that SP 600125, a JNK inhibitor, attenuates hoiamide A-induced neurotoxicity but not that of the AZAs [33]. Hybrid cell death has also been reported previously in neocortical neurons for the Na^+^, K^+^-ATPase inhibitor, ouabain, which produces simultaneous neurotoxicity through both necrosis and apoptosis [34,35,36]. While hoiamide A inhibition of the Na^+^, K^+^-ATPase cannot presently be ruled out, one difference between the neurotoxic response to hoiamide A and ouabain is that nifedipine afforded protection from ouabain but not hoiamide A [35]. Both hoiamide A and ouabain produce an increase in intracellular sodium concentration [22,34,35,36], however, hoiamide A-induced sodium influx is in the micromolar range which is three orders of magnitude less potent than the hoiamide A neurotoxicity. Therefore the neurotoxicity is unlikely to be derived from the increase in the intracellular sodium concentration.

We have previously demonstrated that hoiamide A is a modest affinity (low micromolar) sodium channel partial agonist [22]. Compared to this action on VGSCs hoiamide A is three orders of magnitude more potent in producing LDH efflux, caspase activation and nuclear condensation. Moreover, TTX was without influence on hoiamide A-induced LDH efflux, therefore excluding VGSCs as the molecular target for these actions. We previously found that hoiamide A suppressed spontaneous Ca^2+^ oscillations in primarily cultured neocortical neurons at sub-micromolar concentrations that was independent of an interaction with VGSCs [23]. Whether this action of hoiamide A is involved in the neocortical neuron death observed here is unknown; however, suppression of Ca^2+^ oscillations is not responsible for the hybrid cell death observed for AZAs [33]. 

Our results here also demonstrated that treatment with hoiamide A produced a robust increase in caspase-3 activation. Additionally, Z-VAD-FMK, a permeable, broad-spectrum caspase inhibitor, produced a nearly complete suppression of both hoiamide A-induced LDH efflux and nuclear condensation. These data therefore demonstrate that the caspase cascade is intimately involved in hoiamide A-induced neurotoxicity. This is consistent with the demonstration in which the caspase inhibitor, Z-VAD-FMK, afforded neuroprotection against hybrid neuronal cell death induced by ouabain [35] and the damage in the regions of thalamus and cortex following focal ischemia [37].

Mitogen activated protein kinase signal transduction pathways are among the most widespread mechanisms of eukaryotic cell regulation [38]. Increasing evidence implicates that the JNK pathway plays a role as an important mediator of cell death in many cell populations. Multiple intracellular and extracellular signals are responsible for JNK activation in neuronal cells [39]. Our study of the involvement of MAPK pathways demonstrated that JNK, but not ERK or p38 inhibitors, produced an attenuation of both hoiamide A-induced LDH efflux and nuclear condensation. Additional evidence for the involvement of the JNK signaling pathway was obtained from the demonstration that hoiamide A stimulated JNK phosphorylation in a time dependent manner. 

In summary, we extend the previous finding that hoiamide A is a sodium channel partial agonist, by demonstrating that hoiamide A produces neurotoxicity in neocortical neurons with nanomolar potency. This neurotoxicity is independent of the activation of VGSCs. Moreover, we have demonstrated that hoiamide A-induced neurotoxicity occurs as a result of both necrotic and apoptotic processes. Although the molecular target of hoiamide A-induced neurotoxicity remains to be established, our results demonstrate that the observed neurotoxicity is dependent on both caspase-3 and JNK activation. The potent neurotoxicity and complex neuronal death profile of hoiamide A represents a novel neuronal chemotype from marine cyanobacteria. 

## 4. Materials and Methods

### 4.1. Materials

Trypsin, penicillin, streptomycin, heat-inactivated fetal bovine serum, horse serum and soybean trypsin inhibitor were obtained from Atlanta Biologicals (Norcross, GA, USA). Minimum essential medium, deoxyribonuclease (DNase), poly-l-lysine, cytosine arabinoside, Hoechest-33342, (+)-5-methyl-10,11-dihydro-5H-dibenzo[a,d]cyclohepten-5,10-imine maleate (MK-801), 2,3-dihydroxy-6-nitro-7-sulfamoyl-benzo[f]quinoxaline-2,3-dione (NBQX), 2-[2-[4-(4-Nitrobenzyloxy)phenyl]ethyl]isothiourea mesylate (KBR-7943), nifedipine, fluorescein diacetate, propidium iodide, dichloromethane and polyvinylpyrrolidone were from Sigma (St. Louis, MO, USA). Tetrodotoxin (TTX) was purchased from Tocris Cookson, Inc. (Ellisville, MO, USA). Anti-stress-activated protein kinase/c-Jun N-terminal kinase (SAPK/JNK) antibody and anti-phospho-SAPK/JNK (Thr183/Tyr185) were from Cell Signaling Technology (Danvers, MA, USA). Anthra[1-9-cd]pyrazol-6(2H)-one (SP 600125), 4-(4-Fluorophenyl)-2-[4-(methylsulfinyl) phenyl]-5-(4-pyridyl) 1H-imidazole (SB 203580), 1,4-diamino-2,3-dicyano-1,4-bis (2-aminophenylthio) butadiene (U0126) and *N*-benzyloxycarbonyl-Val-Ala-Asp fluoromethyl ketone (Z-VAD-FMK) were from Biomol International (Plymouth Meeting, PA, USA).

### 4.2. Neocortical Neuron Culture

Primary cultures of neocortical neurons were obtained from embryonic day 16–17 Swiss–Webster mice and processed as previously described [40]. Briefly, pregnant mice were euthanized by CO_2_ asphyxiation and embryos were removed under sterile conditions. Neocortices were collected, stripped of meninges, minced by trituration with a Pasteur pipette and treated with trypsin for 25 min at 37 °C. The cells were then dissociated by two successive trituration and sedimentation steps in soybean trypsin inhibitor and DNase containing isolation buffer, centrifuged and resuspended in Eagle’s minimal essential medium with Earle’s salt (MEM) supplemented with 1 mM l-glutamine, 10% fetal bovine serum, 10% horse serum, 100 I.U./mL penicillin and 0.10 mg/mL streptomycin, pH 7.4. Cells were plated onto poly-l-lysine-coated 96-well clear-bottomed black-well culture plates (Corning Life Science, Acton, MA, USA) at a density of 1.5 × 10^5^ cells/well. Cells were incubated at 37 °C in a 5% CO_2_ and 95% humidity atmosphere. Cytosine arabinoside (10 μM) was added to the culture medium after 24–48 h post plating to prevent the proliferation of nonneuronal cells. The culture medium was changed on 5, 7, and 9 days *in vitro* (DIV) using Neurobasal medium supplemented with B-27, 0.2 mM l-glutamine, 100 I.U./mL penicillin and 0.10 mg/mL streptomycin, pH 7.4. All animal use protocols were approved by the Creighton University Institutional Animal Care and Use Committee (IACUC).

### 4.3. Lactate Dehydrogenase (LDH) Activity Assay

Neocortical neurons cultured in 96-well plates were used for LDH efflux assay as described previously [33]. All assays were carried out in the presence of 0.1% dimethyl sulfoxide (DMSO), which by itself had no effect on LDH efflux in neocortical neurons. Neocortical neurons (8–10 DIV) were continuously exposed to the desired concentrations of hoiamide A in the culture medium. The medium was then collected and assayed for LDH activity. LDH activity was determined spectrophotometrically as described by [41]. Toxicity data are reported as LDH activity units per plate. 

### 4.4. Hoechst 33342 Staining

Neocortical neurons cultured in 96-well plates (8–10 DIV) were exposed to hoiamide A for desired times. The medium was then aspirated and a concentration of 1.6 μg/mL of Hoechst 33342 dye (cell permeable) in Locke’s buffer (in mM: 8.6 Hepes, 5.6 KCl, 154 NaCl, 5.6 Glucose, 1.0 MgCl2, 2.3 CaCl2, 0.0001 glycine, pH 7.4) was added to the neocortical neurons. The neurons were then incubated for 15 min at 37 °C in the dark. Images were then taken using an Olympus IX71 confocal microscope. 

### 4.5. Caspase-3 Activity Assay

Neocortical neurons cultured in 96-well plates (DIV 8) were treated with hoiamide A for the desired time. The cells were then washed three times with PBS and lysed in 60 μL lysis buffer (20 mM Tris, 100 mM NaCl, 0.5 mM EDTA, 0.01% Triton X-100, pH 7.5). After centrifugation for 10 min at 5000× *g*, the supernatant (40 μL) was mixed with equal volume of 2× reaction buffer (20 mM PIPES, 4 mM EDTA, 0.2% CHAPS, 10 μM DTT, pH: 7.4) containing 25 μM of a fluorometric substrate, bisamide derivative of rhodamine 110 (Z-DEVD-R110), and incubated in the dark for 20 min at RT. The fluorescent products, monoamide R110 (R110) were determined using a FLIPR I (Molecular Devices, Sunnyvale, CA, USA) by measuring emission fluorescence at 525 nm with 480 nm excitation. Caspase-3 activity was reflected by the cleavage of Z-DEVD-R110 to the fluorescent product, R110.

### 4.6. Western Blotting

The western blotting experiments were performed as described previously [33]. Briefly, cultures were treated with 30 nM of hoiamide A and incubated at 37 °C for specified times in the culture medium. Cultures were then transferred to an ice slurry to terminate treatment. After washing with ice cold PBS, cells were harvested in ice-cold lysis buffer containing 50 mM Tris, 50 mM NaCl, 2 mM EDTA, 2 mM EGTA, 1% NP-40, 2.5 mM sodium pyrophosphate, 1 mM sodium orthovanadate, 1 μg/mL leupeptin, 1 μg/mL aprotinin, 1 μg/mL pepstatin and 1 mM phenylmethylsulfonyl fluoride just prior to use and incubated for 20 min at 4 °C. Cell lysates were centrifuged at 16,000× *g* for 10 min at 4 °C. The supernatant was assayed by the Bradford method to determine protein content. Equal amounts (30 μg) of protein were mixed with the Laemmli sample buffer and boiled 5 min. The samples were loaded onto a 12% SDS-PAGE gel and transferred to a nitrocellulose membrane by electroblotting. The membranes were blocked in TBST buffer (20 mM Tris, 150 mM NaCl, 0.1% Tween 20) with 5% skimmed milk for 1 h at room temperature. After blocking, membranes were incubated overnight at 4 °C in primary antibody diluted in TBST buffer containing 5% skimmed milk. The blots were washed and incubated with the secondary antibody conjugated with horseradish peroxidase for 1 h, washed four times in TBST buffer and exposed with ECL Plus for 3 min. Blots were exposed to HyBlot CL film (Denville Scientific, Inc., Metuchen, NJ, USA) and developed. Membranes were stripped with stripping buffer (63 mM Tris base, 70 mM SDS, 0.0007% 2-mercaptoethanol, pH = 6.8) and reblotted for further use. 

### 4.7. Fluorescein Diacetate (FDA) and Propidium Iodide (PI) Staining

The FDA/PI staining was performed as described by Jones and Senft [42] to quantify neuronal death. Briefly, neocortical neurons (8–10 DIV) cultured in 12-well plate were treated with vehicle or hoiamide A for 24 h. After washing 3X using PBS buffer, 1 mL of PBS buffer containing FDA (5 μg/mL) and PI (1 μg/mL) was add to each well and incubated at RT for 5 min. The dye buffer was then aspirated and washed 3X using PBS. Images were taken using an Olympus IX71 fluorescent microscope by using FITC filter (FDA) and Texas Red filter (PI) and the cells were counted automatically using Imaris software (Version 6.4, Bitplane Inc., Saint Paul, MN, USA).

### 4.8. Diolistic Labeling

A Helios Gene Gun System (Bio-Rad, Hercules, CA, USA) was used to deliver DiI coated tungsten particles (1.3 µM) (Bio-Rad, Hercules, CA, USA) into paraformaldehyde fixed neocortical neurons as described previously [27]. Briefly, 2.5–3.5 mg of DiI (Invitrogen, Carlsbad, CA, USA) was dissolved in 200 µL of dichloromethane and added over 35 mg tungsten particles. The dye-coated particles were chopped to fine particles and re-suspended in 3 mL deionized water and sonicated for 10 min. After adding 100 µL of polyvinylpyrrolidone (PVP) stock solution (0.96% PVP in ethanol), the suspension was drawn into a PVP pre-coated tefzel tubing and settled for 20–30 min. The tubing was then allowed to dry for 5 min before cutting into bullets using a tube cutter. The neocortical neurons grown on cover slips were shot post-fixation (1.5% paraformaldehyde) using DiI bullets loaded into a Helios Gene Gun and incubated at RT overnight. The cover slips were then mounted on slides and the images were taken on an Olympus IX 71 inverted microscope with a CCD camera controlled by Slidebook software (Intelligent Imaging Innovations, Inc., Denver, CO, USA). To reduce the effect of paracrine neurotrophic factors on neurite growth, only those neurons that were separated from surrounding cells by approximately 150 µm were digitally acquired and analyzed. All neurites in a single neuron including those from secondary branches were manually traced, and the length was measured by Imaris software (Version 6.4, Bitplane Inc., Saint Paul, MN, USA). Total neurite length was calculated by summing all the neurite lengths traced and measured on individual neurons. 

### 4.9. Data Analysis

Concentration-response curves were analyzed by non-linear regression analysis with the GraphPad Prism software (Version 4.0, San Diego, CA, USA). Statistical significance was determined by an ANOVA and, where appropriate, a Dunnett’s Multiple Comparison Test (GraphPad Prsim software, San Diego, CA, USA).

## 5. Conclusions

In the present study, we demonstrated that nanomolar concentrations of hoiamide A triggered a unique neuronal death profile involving both necrotic and apoptotic mechanisms that may derive from an interaction with a common molecular target. Pharmacological evaluation demonstrated that hoiamide A-induced neuronal death required both JNK and caspase signaling pathways. The potent neurotoxicity and unique neuronal death profile of hoiamide A represents a novel neurotoxic chemotype from marine cyanobacteria. 

## Supplementary Files

Supplementary File 1
