# Supplementary material for: Involvement of JNK and Caspase Activation in Hoiamide A-Induced Neurotoxicity in Neocortical Neurons
Source: Mar Drugs. 2015 Feb 10;13(2):903–19. doi: 10.3390/md13020903 (PMC4344608; doi:10.3390/md13020903)
Supplement: Supplementary File 1 [file marinedrugs-13-00903-s001.pdf]

Supplementary Information

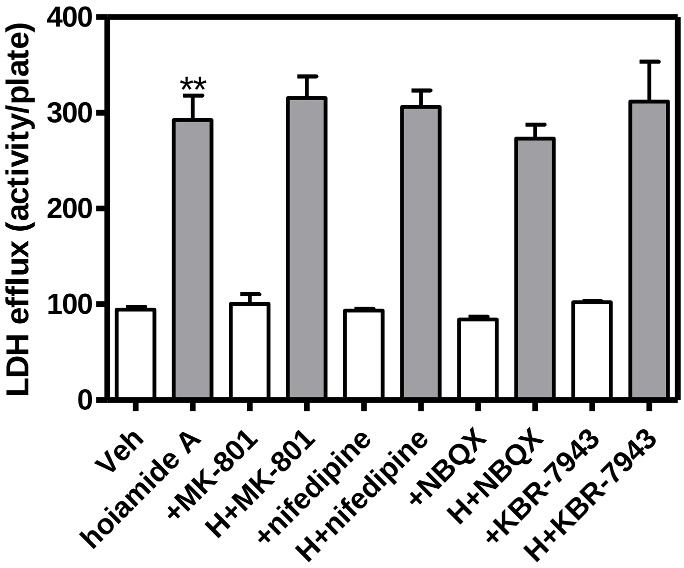

Figure S1. Lack of effect of MK-801 (1 μM), nifedipine (1 μM), NBQX (1 μM) and KBR-7943 (3 μM) on 30 nM hoiamide A-induced LDH efflux. These data were obtained from two independent cell cultures each performed in triplicate (** $p < 0.01$, hoiamide A *vs.* vehicle).

© 2015 by the authors; licensee MDPI, Basel, Switzerland. This article is an open access article distributed under the terms and conditions of the Creative Commons Attribution license (http://creativecommons.org/licenses/by/4.0/).
